# Reduced insulin/IGF-1 signaling and loss of *pals-22* promote repetitive DNA silencing in *C. elegans*

**DOI:** 10.17912/micropub.biology.001747

**Published:** 2025-08-06

**Authors:** Chee Kiang Ewe, Guy Teichman, Oded Rechavi

**Affiliations:** 1 School of Neurobiology, Biochemistry and Biophysics, Wise Faculty of Life Sciences & Sagol School of Neuroscience, Tel Aviv University, Tel Aviv, Tel Aviv, Israel

## Abstract

Non-coding small RNAs and Argonaute proteins mediate conserved defenses against foreign genetic elements. *C. elegans* mutants in the insulin/IGF-1 signaling (IIS) have previously been shown to exhibit an enhanced response to exogenous RNAi. Here, we found that the loss of IIS via *daf-2* enhances transgene silencing, which is reversed by knocking out *daf-16 /foxO* . Similarly, *pals-22* mutants show enhanced RNAi and upregulation of antiviral RNAi pathway. *daf-2* and *pals-22* mutations exhibit additive effects, and loss of *daf-16* restores transgene expression in *daf-2* mutants but not in *pals-22* mutants, suggesting that these genes act in parallel. RNAi gene expression in *daf-2* mutants lacked a consistent pattern, suggesting IIS may regulate RNAi components via post-translational mechanisms.

**Figure 1. Knocking out IIS pathway promotes repetitive transgene silencing:**
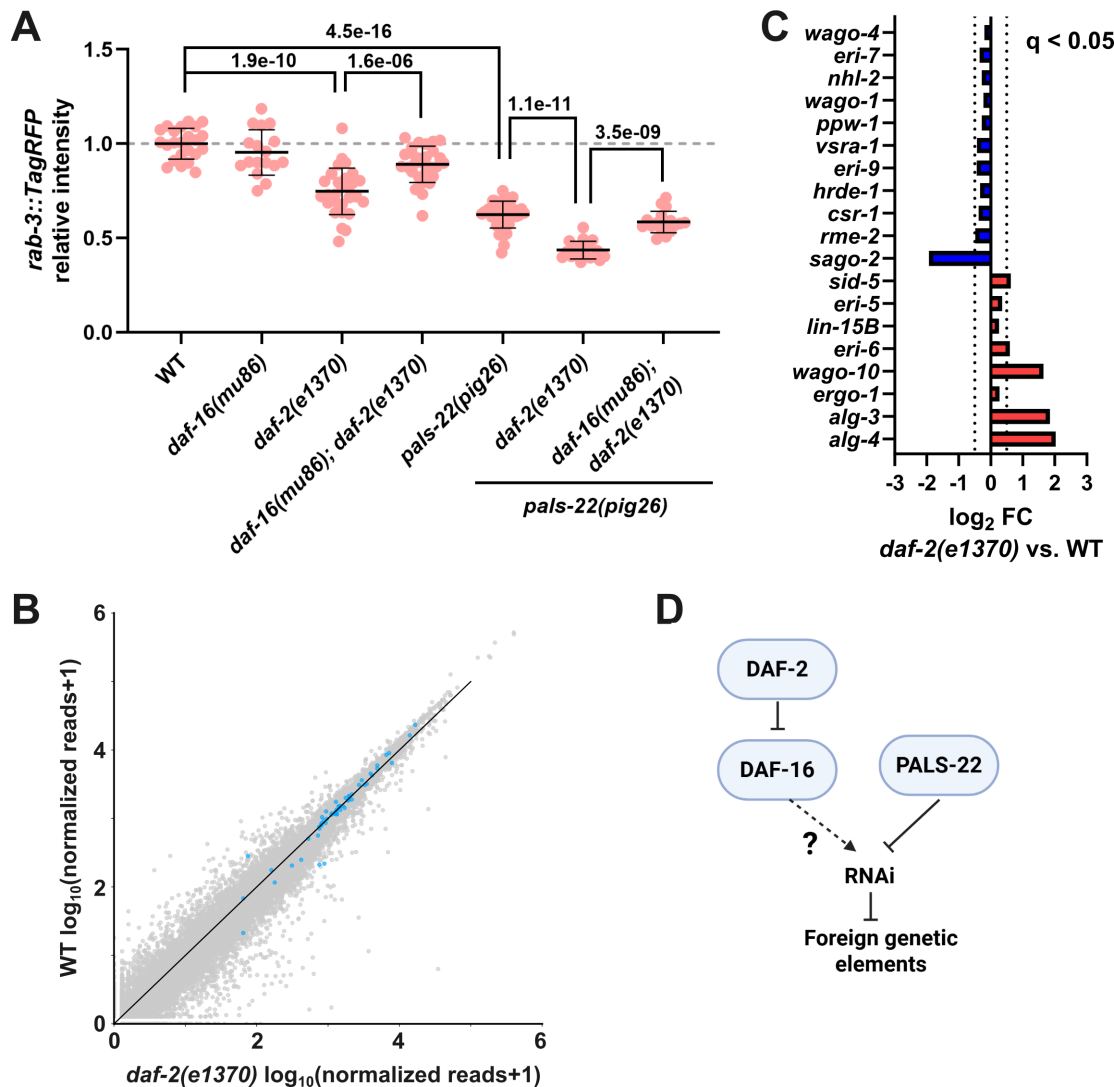
(A) Expression levels of the repetitive transgene *otIs356 [rab-3p(prom1)::2xNLS::TagRFP]* across various genetic backgrounds, as indicated on the x-axis. Each data point represents an individual animal. Statistical significance was determined by non-parametric Kruskal–Wallis test followed by pairwise Wilcoxon Rank Sum tests with Benjamini–Hochberg correction. Numbers indicate q-values. Error represents mean +/- SD. (B) Scatter plot showing gene expression in wild-type and *daf-2 (1370)* mutants. The read counts were normalized using Relative Log Expression method. 62 genes with known roles in RNAi regulation are highlighted in blue. (C) Differential expression of RNAi-related genes in *daf-2 ( e1370 )* mutants relative to wild type (q < 0.05, DESeq2). Dashed lines denote log₂ fold-change thresholds of +/- 0.5. (D) A model illustrating the roles of insulin/IGF-1 signaling and PALS-22 in regulating transgene silencing.

## Description

Non-coding small RNAs, in association with Argonaute proteins (AGO), play an evolutionarily conserved role in defending against foreign genetic elements and mediating antiviral immunity (Maillard et al., 2019; Mello and Conte, 2004). For the past three decades, *Caenorhabditis elegans* has been pivotal in uncovering the mechanisms and regulation of the RNAi pathways. Genetic screens in *C. elegans* have identified many mutants with an enhanced response to double-stranded RNA (dsRNA) (Fischer et al., 2013; Kennedy et al., 2004; Kim et al., 2005). Notably, most Enhanced RNAi (Eri) mutants exhibit increased silencing of multicopy repetitive transgenes, which are recognized by the RNAi machinery as foreign elements, analogous to viruses, and are thus targeted for silencing (Félix and Wang, 2019).

The Ruvkun lab has previously showed that loss of IIS increases the animals' response to dsRNA (Wang and Ruvkun, 2004). Moreover, reduced IIS has been shown to restore germline immortality in PIWI mutants by promoting germline genome surveillance mechanisms (Simon et al., 2014), suggesting a role of IIS in regulating small RNA pathways. Insulin/IGF-1 receptor DAF-2 regulate the activity of FoxO transcription factor DAF-16 , the major downstream effector of the IIS pathway (Murphy and Hu, 2013). In this study, we show that *daf-2 ( e1370 )* mutants exhibit enhanced silencing of repetitive transgenes (pan-neuronal *otIs356 [rab-3p(prom1)::2xNLS::TagRFP]* ), an effect that is suppressed by loss of *daf-16* ( Figure 1A ), indicating that DAF-2 normally acts to suppress the expression of foreign genetic elements in DAF-16-dependent manner.

In a recent study, we showed that loss of PALS-22 , a negative regulator of the intracellular pathogen response that protects against viral and microsporidian infections, leads to upregulation of the antiviral RNAi pathway and the AGO gene *vsra-1* , which contributes to transgene silencing (Ewe et al., 2025). Here, we found that *pals-22* and *daf-2* mutations have an additive effect on transgene silencing (two-way ANOVA, *pals-22* and *daf-2* effects p < 0.0001, interaction effect p = 0.27), and that loss of *daf-16* restores transgene expression in *daf-2 ( e1370 )* mutants, but not in *pals-22* mutants ( Figure 1A ). These results suggest that PALS-22 and the IIS pathway act in parallel to regulate foreign genetic elements.

To investigate how loss of *daf-2* affects the small RNA machinery, we examined the expression of known RNAi pathway regulators (n = 62) in *daf-2 ( e1370 )* mutants ( Figure 1B ); however, we did not observe a clear expression pattern that could account for the strong transgene silencing phenotype in this mutant. For example, *eri* genes and components of the endogenous siRNA pathway showed both up- and downregulation, with no consistent trend ( Figure 1C ). Unlike PALS-22 , which suppresses RNAi and AGO gene expression, it is unlikely that IIS functions through the same mode of regulation. Instead, DAF-2 may regulate RNAi components post-translationally, potentially affecting small RNA amplification and/or their loading onto AGOs, which are critical for robust gene silencing ( Figure 1D ).

## Methods

**Worm cultivation**

*C. elegans* strains were maintained at 20 ^o^ C on Nematode Growth Media (NGM) seeded with *E. coli* OP50 . Genetic crosses were performed using standard procedure, and genotyping primers are provided below.

**Imaging and fluorescence quantification**

To examine transgene silencing, we analyzed the expression of the pan-neuronally expressed, repetitive transcriptional reporter *otIs356 [rab-3p(prom1)::2xNLS::TagRFP]* . Animals were immobilized using 5 mM levamisole and mounted on 2% agarose pads for imaging. Fluorescence images were acquired using either an Olympus IX83 motorized inverted wide-field microscope or an Olympus BX63 motorized upright wide-field microscope. Transgene expression was quantified using Fiji/ImageJ software. To prevent bias, genotype information was blinded from the investigator using the DoubleBlind tool ( https://github.com/GuyTeichman/DoubleBlind ). Corrected total fluorescence per worm was calculated as: integrated density – (area of the selected region × mean background fluorescence). Worms that overlapped with others or were located at the edges of the image were excluded from the analysis.

**Bioinformatics analysis**

All bioinformatics analysis was perform using RNAlysis (Teichman et al., 2023). RNA-seq data was obtained from GEO: GSE271972. Sequencing reads were pseudo-aligned using Kallisto (Bray et al., 2016). We performed differential expression analysis using DESeq2 (Love et al., 2014).

## Reagents

**Table:** 

Strain	Genotype	Source
BFF359	*daf-2 ( e1370 ) III; otIs356 [rab-3p(prom1)::2xNLS::TagRFP] V *	This study
BFF360	*pals-22 ( pig26 ) daf-2 ( e1370 ) III; otIs356 [rab-3p(prom1)::2xNLS::TagRFP] V *	This study
BFF370	*daf-16 ( mu86 ) I; daf-2 ( e1370 ) III; otIs356 [rab-3p(prom1)::2xNLS::TagRFP] V *	This study
BFF371	*daf-16 ( mu86 ) I; pals-22 ( pig26 ) daf-2 ( e1370 ) III; otIs356 [rab-3p(prom1)::2xNLS::TagRFP] V *	This study
BFF151	*pals-22 ( pig26 ) III; otIs356 [rab-3p(prom1)::2xNLS::TagRFP] V. *	This study
BFF369	*daf-16 ( mu86 ) I; otIs356 [rab-3p(prom1)::2xNLS::TagRFP] V *	This study
OH10690	*otIs356 [rab-3p(prom1)::2xNLS::TagRFP] V. *	CGC

**Table:** 

Primer name	Sequence	Description
EE17	GCGACTCGACCTATCAGTGC	*pals-22 ( pig26 )* forward
EE18	ATTTTGCCGCCCATCCCTAA	*pals-22 ( pig26 )* reverse
DAF2 F	CCGACGTTCCGAATCACTCTGAACCTCGACG	*daf-2 ( e1370 )* forward
DAF2 R	GCACAGATTTGTGATGGTATGGCGTACCTGG	*daf-2 ( e1370 )* reverse
EE161	CACCACTCAACTCGAGTCCC	*daf-16 ( mu86 )* forward
EE162	AAAAGCTCACTCCGAAGGAA	*daf-16 ( mu86 )* reverse
